# Enhanced atherosclerosis molecular imaging and therapy with collagen hybridizing peptide functionalized albumin nanoparticles

**DOI:** 10.1186/s12951-025-03721-3

**Published:** 2025-10-28

**Authors:** Jianan He, Jianxun Cai, Jingjing Zhang, Yang Li, Kunpeng Wei, Chenshu Liu, Leye Yan, Xinyan Hu, Lin Huang, Hairun Gan, Dashuai Wang, Bing Li, Huitao Zhang, Pengfei Pang

**Affiliations:** 1 Center for Interventional Medicine, The Fifth Affiliated Hospital, Sun Yat-sen University, Zhuhai, 519000 P. R. China; 2 Guangdong Provincial Engineering Research Center of Molecular Imaging, The Fifth Affiliated Hospital, Sun Yat-sen University, Zhuhai, 519000 P. R. China; 3 Guangdong-Hong Kong-Macao University Joint Laboratory of Interventional Medicine, The Fifth Affiliated Hospital, Sun Yat-sen University, Zhuhai, 519000 P. R. China; 4 Department of Ophthalmology, The Fifth Affiliated Hospital, Sun Yat-sen University, Zhuhai, 519000 P. R. China; 5 Department of Critical Care Medicine, The Fifth Affiliated Hospital, Sun Yat-sen University, Zhuhai, 519000 P. R. China

**Keywords:** Atherosclerosis, Collagen degradation, Collagen hybridizing peptide, Albumin nanoparticles, Photoacoustic imaging, Paclitaxel

## Abstract

Intraluminal drug coated devices, such as paclitaxel (PTX)-coated balloons, are commonly used to treat arterial occlusive diseases caused by atherosclerosis. However, their efficacy is limited by drug loss, poor drug retention, insufficient penetration and high costs. Nanoparticles with molecular targeting capabilities offer a promising solution to these challenges. Collagen hybridizing peptide (CHP) specifically binds to degraded collagen in atherosclerotic plaques, enabling precise molecular imaging and targeted therapy. In this study, we demonstrated that collagen degradation was significantly elevated in atherosclerotic plaques and could be effectively identified by CHP in both atherosclerosis patients and mice models. Using the emerging copper-free click chemistry reaction, we functionalized the widely used albumin-based indocyanine green nanoparticles and albumin-bound paclitaxel nanoparticles with CHP, developing a targeted nanoplatform for molecular imaging and therapy. In vivo photoacoustic (PA) imaging and ex vivo fluorescence (FL) imaging revealed significantly enhanced PA and FL signals in carotid artery and aorta plaques with CHP functionalized nanoparticles engaging. Furthermore, therapeutic evaluations showed that CHP significantly improved the plaque-targeting capability of albumin-bound paclitaxel nanoparticles, thereby enhancing their therapeutic efficacy against atherosclerotic plaques. These findings highlighted CHP-functionalized albumin nanoparticles as a promising strategy for precise and effective atherosclerosis molecular imaging and treatment.

**Graphical Abstract:**

**Figure:**
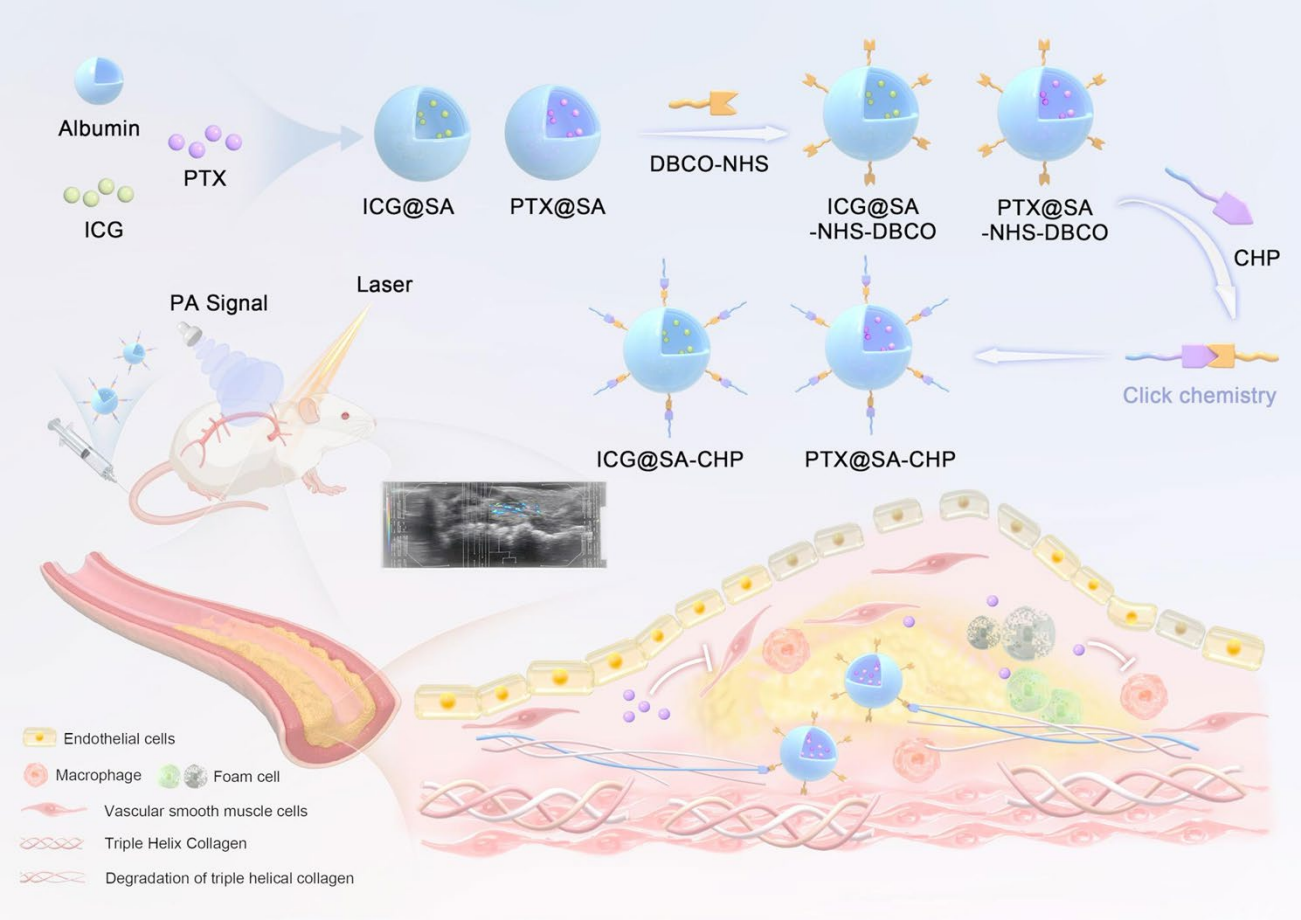
Albumin nanoparticles functionalized with CHP by copper-free click chemistry reaction, enhancing the imaging ability and therapeutic efficacy for atherosclerosis by elevated plaque targeting capability.

**Supplementary Information:**

The online version contains supplementary material available at 10.1186/s12951-025-03721-3.

## Highlights

1 - Collagen hybrid peptide (CHP) specifically binds to degraded collagen in atherosclerotic plaques.

2 - Copper-free click chemistry reaction achieves efficient CHP conjugation to albumin nanoparticles.

3 - CHP functionalized albumin-based indocyanine green nanoparticles precisely target degraded collagen in atherosclerosis mice model, enabling accurate in vivo photoacoustic imaging of plaques.

4 - CHP markedly enhance targeted delivery and topical paclitaxel concentration of clinically used albumin-bounded paclitaxel in atherosclerotic plaques.

5 - CHP functionalized albumin-bounded paclitaxel nanoparticles significantly improved therapeutic efficacy of atherosclerosis without notable toxicity.

**Supplementary Information:**

The online version contains supplementary material available at 10.1186/s12951-025-03721-3.

## Introduction

Atherosclerosis is a leading cause of cardiovascular disease, including myocardial infarction, stroke, and peripheral artery disease, often resulting in arterial occlusion, which can lead to mortality and disability [1]. In routine clinical practice, imaging techniques such as computed tomography angiography (CTA) and digital subtraction angiography (DSA) rely on contrast agents to determine the site of atherosclerotic plaque [2]. However, these methods have several disadvantages, including renal impairment, calcification artifacts and potential allergic reactions, restricting their widespread of clinical application [3, 4]. Endovascular therapy remains the primary treatment for patients with atherosclerosis induced arterial occlusive diseases.

However, it faces significant challenges, including its inability to halt the progression of atherosclerotic plaques, restenosis after treatment, and other factors contributing to treatment failure [5]. Recently, drug coated devices, particularly drug-coated balloons (DCBs) coated with antiproliferative drugs such as paclitaxel, have been developed to address these issues. These devices deliver drugs to the lesion site during balloon expansion, inhibiting smooth muscle cell proliferation and reducing inflammation, thereby exerting therapeutic effects on atherosclerotic plaques [6]. Nevertheless, DCBs still encounter several limitations, including ineffective transmural drug distribution, difficulty in controlling tissue-retained dosage and residence time, requirement of a high initial drug loading, drug loss into the systemic circulation, risk of distal drug embolization and high costs. These challenges hinder DCBs from achieving optimal therapeutic efficacy [7–9]. Therefore, developing novel molecular imaging and therapeutic nanoparticles with high targeting ability is essential to improve the precision and effectiveness of atherosclerosis treatment.

As the main component of the extracellular matrix, collagen synthesis and degradation are crucial in the development and progression of atherosclerosis [10, 11]. Early studies have shown that degraded collagen can promote vascular smooth muscle cells (VSMCs) proliferation and migration from the media to the intima, thereby accelerating the formation and progression of atherosclerotic plaques [12]. Furthermore, collagen degradation may destabilize the fibrous cap, increasing the risk of plaque rupture and life-threatening vascular events such as myocardial infarction, cerebral infarction, and extremity arterial embolism [13, 14]. Previously, we have synthesized the collagen hybridizing peptide (CHP) which could mimics the triple-helical structure of native collagen and specifically bind to degraded collagen by hybridize with the unfolded collagen strands, thereby re-forming a triple helical structure to detect collagen degradation both in vitro and in vivo [15–17]. As a synthetic peptide, CHP can be functionalized for targeted imaging and therapy by conjugating it with imaging and therapeutic agents [18, 19].

In this study, we demonstrated that CHP could effectively identify atherosclerotic lesions in both human tissue and animal models. We then constructed targeted molecular imaging and therapeutic nanoparticles by conjugating CHP with albumin nanoparticles encapsulating either indocyanine green or paclitaxel. This was achieved by click chemistry reaction involving azide groups from CHP peptides, DBCO-Sulfo-NHS linker, and amidocyanogen groups from albumin. The efficacy of CHP functionalized nanoparticles in atherosclerosis imaging and therapy was evaluated through in vivo photoacoustic imaging and therapeutic studies. Our results indicated that CHP functionalized albumin nanoparticles could provide real-time observation of atherosclerotic lesions and significantly enhance the therapeutic effect of paclitaxel in treating atherosclerosis. These findings highlighted the potential of CHP functionalized nanoparticles as a promising strategy for precise molecular imaging and targeted treatment of atherosclerosis.

## Materials and methods

### Human tissues

Human artery tissues were obtained from patients undergoing amputation at the Fifth Affiliated Hospital of Sun Yat-sen University and approved by the Ethics Committee of the Fifth Affiliated Hospital of Sun Yat-sen University. Atherosclerotic plaque samples were collected from patients with severe atheromatous occlusion, while normal artery tissues were obtained from trauma-related amputations. All tissue samples were histologically examined using Hematoxylin and Eosin staining (H&E), Oil Red O staining (ORO), and Masson’s trichrome staining (Masson Staining). Fluorescence staining with Cy3-labeld CHP (Cy3-CHP) was performed to assess the specificity of CHP in identifying atherosclerotic plaques.

### Mouse model of atherosclerosis

All animal experiments were approved by Experimental Animal Ethics Committee of the Fifth Affiliated Hospital of Sun Yat-sen University and conducted in accordance with NIH guidelines. Male apolipoprotein E-deficient (ApoE-/-) mice, aged 6–8 weeks, were fed with a high-fat diet (HFD) (D12108C, 41% kcal from fat, 43% kcal from carbohydrates and 17% kcal from protein; Xietong Bio) for 12 weeks to induce the formation and aggravation of atherosclerotic plaques. Age-matched male ApoE-/- mice (6–8 weeks) were fed with a normal diet for 12 weeks and served as the control group. At the end of the feeding period, mice were euthanized, and their aortas were harvested for histological staining and fluorescence staining, as described previously.

### Peptide synthesis and labeling

CHP peptides were synthesized using standard Fmoc-chemistry solid-phase synthesis on a TentaGel R RAM resin (substitution level: 0.18 mmol/g). The required Fmoc-Gly-OH, Fmoc-Pro-OH, Fmoc-Hyp-(tBu)-OH, and Fmoc-cis-4-fluoro-Pro-OH were purchased from GL Biochemical Company. O-(7-Azabenzotriazol-1-yl)-N, N, N′, N′-tetra-methyluronium hexafluorophosphate (HATU, Aladdin) and 1-hydroxy-7-azabenzotriazole (HOAt, MACKLIN) were used as the coupling reagents. The Fmoc protecting group was removed by treating the resin with 20% vol/vol piperidine in dimethylformamide (DMF) for 30 min. For each coupling reaction, five molar equivalents of amino acid residues and coupling reagents, along with 7.5 molar equivalents of diisopropylethylamine (DIPEA, Sigma) in DMF, were used over a period of 3 h. Peptide labeling was performed on the resin by reacting it with the N-hydroxysuccinimide ester of Sulfo-Cyanine 3 (Cy3) for over 24 h. The resins were subsequently treated with trifluoroacetic acid (TFA, MACKLIN)/triisopropylhydrosilane (TIS, MACKLIN)/water (95 : 2.5 : 2.5) for 3 h, and the crude peptides were precipitated by adding an excess amount of cold ether to the TFA solution. Purification was performed using reverse phase high-performance liquid chromatography (RP-HPLC) on an Agilent ZORBAX StableBond 300 C18 column, employing a linear gradient mixture of water (0.1% TFA) and acetonitrile (0.1% TFA) (5 − 35% acetonitrile in 30 min) as the mobile phase.

### Fluorescence staining

Human and mice arterial tissues were frozen in optimal cutting temperature compound (OCT compound, Tissue-Tek, Sakura), and cryosections were prepared at a thickness of 8 μm. The frozen slides were incubated in PBS three times for 5 min each to remove the OCT compound. Subsequently, 100 µl of PBS solutions containing 10 µM Cy3-labeld CHP were applied to the tissue sections and incubated overnight at 4 °C. Following the incubation, the slides were washed three times for 5 min each with PBS to remove any unbound peptides. The sections were then stained with DAPI (Beyotime, C1002, 1:1000 dilution in PBS) for 10 min. After DAPI staining, the slides were mounted with antifade mounting medium (P0126, Beyotime) to preserve the fluorescence signals. Finally, the slides were imaged using a fluorescence microscope to assess the binding and localization of CHP.

### Preparation of ICG-bovine serum albumin-CHP nanoparticles (ICG@BSA-CHP NPs) and paclitaxel-human serum albumin-CHP nanoparticles (PTX@HSA-CHP NPs)

Initially, ICG@BSA NPs were prepared following protocol: 40 mg of Bovine serum albumin (BSA, PH0501, Phygene) was dissolved in 4 mL of deionized water, and the PH was adjusted to 8.5 using a NaOH aqueous solution (S111502, Aladdin). 15.5 mg of Indocyanine green (ICG, I107931, Aladdin) was dissolved in 2mL DMSO, then added to 6 mL of acetone (preheated to 40 °C). The ICG solution was combined with the 4 mL BSA aqueous solution all at once, with stirring at room temperature. To stabilize the nanoparticles, 1 mg of 1-ethyl-3-(3-dimethylaminopropyl)-carbodiimide (EDC, BD19757, BIDE) was added to promote cross-linking. Stirring continued at room temperature for 2 h to ensure complete cross-linking of amide bonds. The resulting ICG@BSA NPs suspension was subjected to rotary evaporation to remove acetone, followed by ultrafiltered at 4 °C using a 50 kilodalton (kDa) molecular weight cutoff ultrafiltration tube at 4000 rpm until the filtrate was colorless. The nanoparticles were then stored at 4 °C.

To conjugate CHP to the ICG@BSA NPs, a click chemistry method was employed. The molar equivalent ratio of ICG@BSA NPs to DBCO-Sulfo-NHS ester (A124-100, Click Chemistry Tools) to CHP was set to 1:3:5. First, DBCO-Sulfo-NHS ester was dissolved in 1× PBS to prepare a 10 mM solution. This solution was mixed with the ICG@BSA NPs solution and allowed to react on a shaker at room temperature for 30 min. Unreacted and excess DBCO-NHS were removed using ultrafiltration tubes with a 50 kDa molecular weight cutoff at room temperature. Subsequently, CHP with azide group was added to the ICG@BSA NHS-Sulfo-DBCO solution, and the reaction was carried out on a shaking table at room temperature overnight. Unreacted and excess CHP were removed using ultrafiltration.

The same methods were employed to synthesize PTX@HSA-CHP NPs, in which CHP was conjugated to PTX@HSA NPs (Selleck, Cat#E1068) via a click chemistry reaction. All NPs were sterilized by 0.22 μm membrane filters to remove microorganisms before the intravenous administration.

### Characterization of the nanoparticles

The characterization of NPs was conducted to evaluate their physicochemical properties, including particle size, zeta potential, morphology, UV-Vis-NIR absorption, and physical stability.

#### Morphology characterization, particle size and zeta potential

The morphology of the NPs (ICG@BSA-CHP NPs, ICG@BSA NPs, PTX@HSA-CHP NPs, and PTX@HSA NPs) was examined using transmission electron microscopy (TEM). Samples were prepared by depositing a small droplet of nanoparticle suspension onto a carbon-coated copper grid, followed by air drying. TEM images were acquired to assess the shape and surface characteristics of the nanoparticles. The mean particle size and zeta potential of the nanoparticles were determined by dynamic light scattering (DLS) (Malvern, UK). Samples were diluted with distilled water to an appropriate concentration and measured three times at 25 °C.

#### UV-Vis-NIR spectral analysis

The UV-Vis-NIR spectra of the ICG@BSA-CHP NPs, ICG@BSA NPs, free ICG, and CHP were recorded using a Synergy™ NEO microplate reader (BioTek, Winooski, VT, USA). Samples were prepared in appropriate solvents and scanned across the relevant wavelength range to determine their optical properties and verify the successful encapsulation of ICG within the NPs.

#### Physical stability

The physical stability of the NPs was assessed by monitoring changes in particle size over time. The particle sizes of PTX@HSA-CHP NPs and PTX@HSA NPs were measured at 1 day, 2 days, 3 days, and 4 days using the DLS method described above. Samples were stored at room temperature and diluted with distilled water prior to each measurement to evaluate any aggregation or degradation over the storage period.

#### In vitro release of Paclitaxel from nanodrugs

To investigate the release profiles of PTX from the HSA NPs, 20 mg of PTX@HSA-CHP NPs and PTX@HSA NPs were suspended in 1 mL of 10 mM PBS (PH 7.4) and dialyzed against 20 mL of 10 mM PBS (PH 7.4) at 37 °C, using semipermeable membranes with a 10 kDa molecular weight cutoff (MWCO) (Spectrum Labs, Rancho Dominguez, CA, USA). At predetermined time points (1, 2, 4, 6, 8, 12, 24, 48, 72, 96 h), 0.3 mL of the dialysate was collected and replaced with an equal volume of fresh PBS. The amount of PTX released into the dialysate was quantified using high performance liquid chromatography (HPLC) (SHIMADZU-LC-40) with a ZORBAX SB-C18 column (250 × 4.6 mm, 5 μm; Agilent Technologies, Palo Alto, CA, USA) at ambient temperature. An isocratic elution was performed at a flow rate of 1.0 mL/min with a mobile phase consisting of 40% solvent A (deionized water, DW) and 60% solvent B (acetonitrile, ACN). The eluates were monitored at a wavelength of 227 nm for PTX detection.

### Biosafety evaluation of ICG@BSA-CHP NPs

To evaluate the biodistribution and potential toxicity of ICG@BSA-CHP NPs, untreated ApoE-/- mice were intravenously injected with 100 µl of ICG@BSA-CHP NPs (800 µM). At 1, 6, 12, and 24 h post-injection, main organs were harvested and imaged using the IVIS imaging system to assess the biodistribution of the nanoparticles. At 1 day, 7 days and 14 days after injection, blood samples and main organs were collected for hematological analysis and hematoxylin & eosin (H&E) staining.

### In vitro and in vivo photoacoustic imaging (PA imaging)

Photoacoustic (PA) imaging was performed using a Vevo LAZR-X photoacoustic imaging system (FUJIFILM Visual Sonics Inc., Toronto, Canada). To evaluate the PA signal of the NPs, ICG@BSA-CHP NPs, ICG@BSA NPs, free ICG and PBS solutions were prepared and placed in fine bore polythene tubes (Smiths Medical) with an inner diameter (ID) of 0.28 mm and outer diameter (OD) of 0.61 mm.PA spectra were acquired using the Spectro module over a wavelength range of 700–950 nm, with measurements taken at 5 nm intervals.

For in vivo PA imaging, atherosclerosis model mice (ApoE-/- mice fed with HFD for 12 weeks) and control model mice (ApoE-/- mice fed with normal diet for 12 weeks) were used. Each group received an intravenous injection of 100 µl of either ICG@BSA-CHP NPs (800 µM), ICG@BSA NPs (800 µM). After 30 min post-injection, the mice were anesthetized and depilated to minimize light scattering, then positioned in the Vevo LAZR-X photoacoustic imaging system. The MX250 transducer (center transmit frequency ≈ 21 MHz) was used to acquire signals, and the multi-wavelength photoacoustic module was applied for imaging. The total observation time for each mouse was controlled to be 1 h to prevent any adverse effects on their health.

### Ex vivo fluorescence imaging (FL imaging)

Following the completion of all PA imaging experiments, the mice were sacrificed and their aortas were carefully dissected for further analysis. The dissected aortas were then subjected to ex vivo FL imaging in Z-view and A-view using an excitation wavelength of 780 nm and an emission wavelength of 845 nm. The imaging was conducted on an IVIS Lumina Ⅲ Imaging System (Caliper Life Sciences, Hopkinton, MA) to evaluate the fluorescence signal and distribution in the atherosclerotic plaques.

### Biosafety evaluation of therapeutic nanoparticles

To evaluate the biodistribution and potential toxicity of PTX@HSA-CHP NPs, untreated ApoE-/- mice were intravenously injected with 100 µl of PTX@HSA-CHP NPs (equivalent to PTX 0.25 mg/kg). At 1, 6, 12, and 24 h post-injection, main organs were harvested and imaged using the HPLC to assess the concentration of PTX. At 1 day, 7 days and 14 days after injection, blood samples and main organs were collected for hematological analysis and hematoxylin & eosin (H&E) staining.

### In vivo targeted delivery capability of therapeutic nanoparticles

To evaluate the targeted delivery capability of the nanoparticles, atherosclerotic (AS) model mice, which had been fed a high-fat diet (HFD) for 6 weeks, were randomly divided into four groups, and treated with 100 µl of saline, PTX, PTX@HSA NPs, and PTX@HSA-CHP NPs solution (equivalent to PTX 0.25 mg/kg), respectively. At 1 h post injection, the mice were euthanized, and their aortic roots and aorta were dissected. Aortic roots samples were collected for immunofluorescence staining to evaluate human serum albumin (HSA) by HSA antibody (SAB4200711, Sigma-Aldrich). Aorta samples containing plaques were collected to assess paclitaxel concentrations by using HPLC.

### In vivo treatment of atherosclerotic plaques

After 6 weeks HFD, ApoE-/- mice were stochastically divided into four groups, and treated with saline, PTX, PTX@HSA NPs, and PTX@HSA-CHP NPs solution (equivalent to PTX 0.25 mg/kg) via tail intravenous injection (once per week). Mice were continued to be fed with HFD throughout the treatment period. At the end of the 6 weeks treatment, mice were euthanized, followed by cardiac perfusion and anatomy of the aortas. ORO staining was performed to visualize and photograph the laden of aortic plaques. Frozen sections of the aortic roots were prepared and stained with ORO, H&E, Masson and immunofluorescence staining (α-SMA and CD68) to assess the aggravation of atherosclerosis.

### Statistic analysis

All the experiments were performed in triplicate or more to ensure reproducibility, and the data were presented as mean ± standard deviation (SD). Statistical analysis was conducted using an unpaired two-tailed Student’s t-test for comparisons between two groups or a one-way analysis of variance (ANOVA) for comparisons among multiple groups with SPSS software (version 27.0). Statistical significance was defined as *P < 0.05; **P < 0.01, and ***P < 0.001. Image analysis was conducted using ImageJ software. In vivo photoacoustic imaging data were processed and analyzed using Vevo Lab software (version 3.2.6). Ex vivo fluorescence imaging data were processed and analyzed using Living Image software (version 4.4). Graphical representations were performed using GraphPad Prism (version 10.1.2).

## Results

### Evaluation of collagen degradation in atherosclerosis

**Fig. 1:**
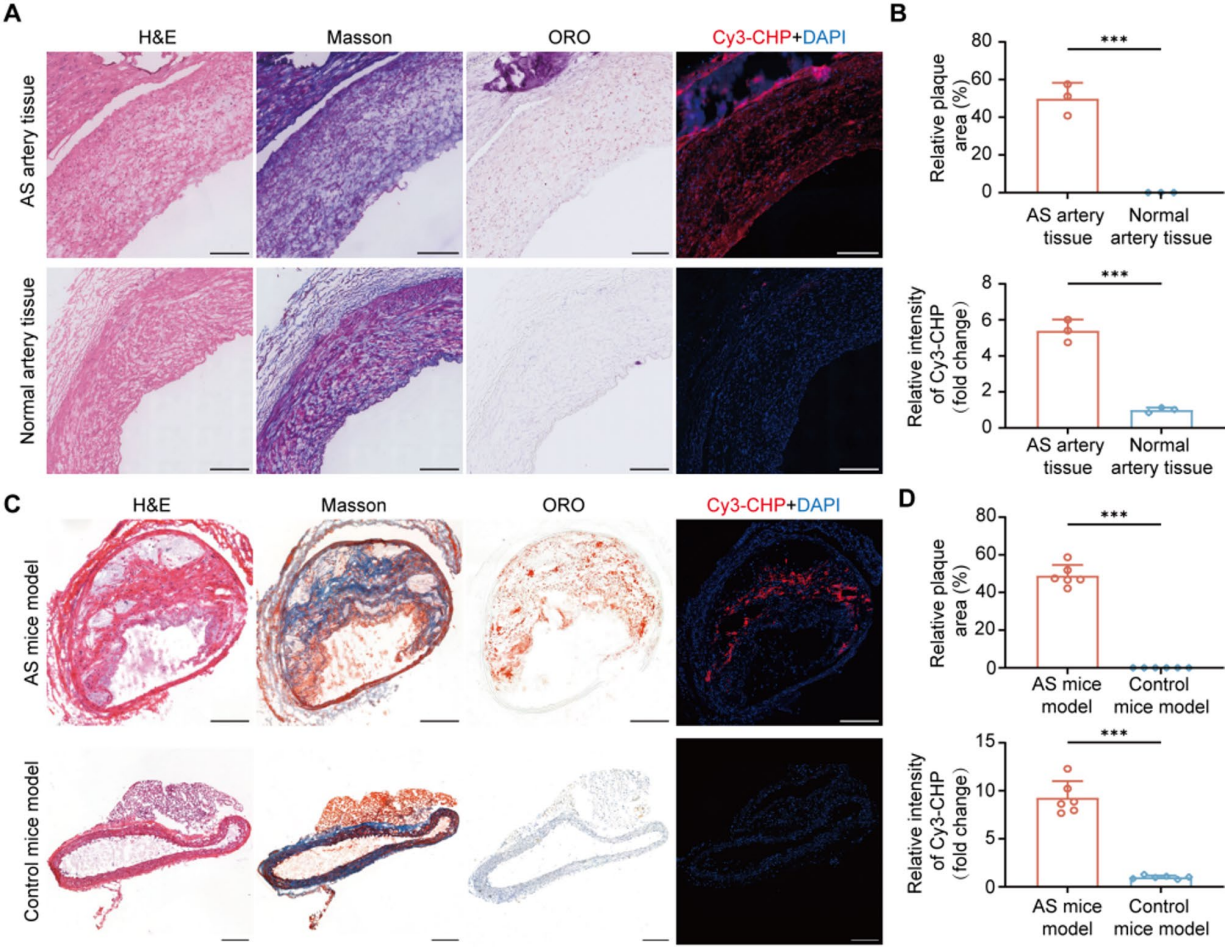
**A**) Representative H&E, ORO, Masson, and fluorescence staining images of atherosclerotic human artery tissues and normal artery tissues. **B**) Quantitative analysis of relative plaque area in the aorta and the relative intensity of Cy3-CHP fluorescence in human artery tissues. **C**) Staining and fluorescence images of atherosclerotic animal model compared to control mice model. **D**) Quantitative analysis of relative plaque area and Cy3-CHP fluorescence intensity in mice models. Experimental data were expressed as mean ± SD. Statistical significance: *P < 0.05, **P < 0.01, ***P < 0.001; ns, no significance. The scale bars were 200 μm

To investigate the in vivo tissue selectivity of CHP in the atherosclerosis, we performed histological staining and fluorescence staining on both human artery tissues and atherosclerosis mice models. As shown in Fig. 1A, histological staining confirmed the presence of atherosclerotic plaques in arteries from AS patients, whereas normal arteries exhibited no significant lipid accumulation. In response, fluorescence staining demonstrated strong Cy3-CHP signals in atherosclerotic plaques with approximately a 5.38 fold increase in fluorescence intensity compared to normal artery tissues (AS arteries vs. Normal arteries, 5.38 ± 0.63 vs. 1.00 ± 0.14, *n* = 3, *P* < 0.001) (Fig. 1B), indicating the presence of degraded collagen. Similarly, in the atherosclerotic mouse model, histological staining further validated the formation of atherosclerotic plaque (Fig. 1C). Fluorescence staining results showed a 9.27 fold increase in Cy3-CHP fluorescence intensity in AS mice model compared to control mice model (AS mice vs. Control mice, 9.27 ± 1.72 vs. 1.00 ± 0.18, *n* = 6, *P* < 0.001) (Fig. 1D), further supporting the selective binding of CHP to the degraded collagen in atherosclerotic plaques. These findings indicated that collagen degradation were significantly enhanced in atherosclerotic plaques. As a peptide probe specifically designed to bind degraded collagen, CHP effectively highlighted the collagen degradation in atherosclerotic plaque. Therefore, CHP held potential as a molecular probe for targeted imaging and therapy of atherosclerotic plaques.

### Preparation of the molecular imaging nanoparticles

CHP, functionalized with an azide group at the end of its peptide chain, was utilized to target atherosclerotic plaques. DBCO-NHS ester served as a linker in the copper-free click chemical reaction, which was used to conjugate CHP to ICG@BSA imaging NPs. This process resulted in the formation of a photoacoustic imaging probe with CHP as the targeting moiety, ICG for photoacoustic imaging, and BSA as the nanocarrier.

**Fig. 2:**
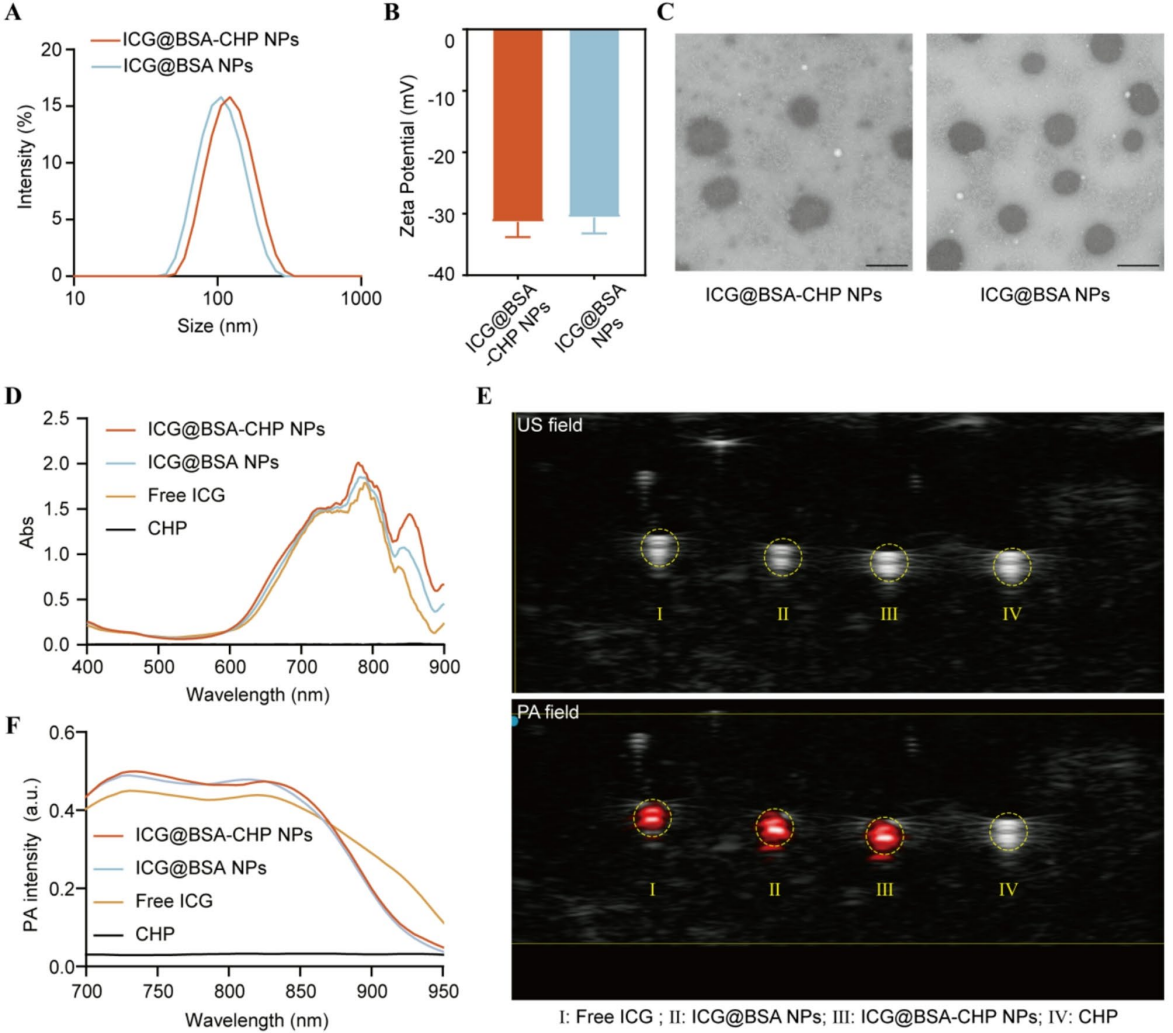
Characterizations of PA imaging albumin NPs. **A**) Particle Size distribution and **B**) Zeta potential of ICG@BSA-CHP NPs and ICG@BSA NPs. **C**) Morphology of NPs observed by TEM. The scale bars were 100 nm. **D**) UV-vis absorption spectroscopy of ICG@BSA-CHP NPs and ICG@BSA NPs. **E**) Ultrasonic (US) and PA image of free ICG, ICG@BSA NPs, ICG@BSA-CHP NPs and CHP solution. **F**) In vitro photoacoustic spectra of free ICG, ICG@BSA NPs, ICG@BSA-CHP NPs and CHP solution at different wavelengths

The particle size of ICG@BSA-CHP NPs and ICG@BSA NPs were determined by DLS as 117 nm (polydispersity index, PDI = 0.105) and 105 nm (PDI = 0.115), respectively. The size distribution curves were presented in Fig. 2A. Meanwhile, the zeta potentials of ICG@BSA-CHP NPs and ICG@BSA NPs were measured at −31.3 mV and − 30.6 mV, respectively (Fig. 2B). Furthermore, transmission electron microscopy (TEM) images confirmed that both nanoparticle types possess uniform, spherical shapes (Fig. 2C).

Ultraviolet-visible (UV-Vis) absorption spectroscopy demonstrated the successful loading of ICG within the albumin NPs, no significant alterations in the absorption profile upon CHP conjugation (Fig. 2D).

To further realize the in vivo imaging of atherosclerotic plaques, PA imaging was conducted. Nanoparticles containing ICG can produce PA signals under pulsed laser irradiation by converting laser energy into heat. Accordingly, PA images of nanoparticles within a tube were acquired, and the corresponding PA spectra were recorded. Figure 2E demonstrated that both ICG@BSA-CHP NPs and ICG@BSA NPs exhibited strong PA signals compared to CHP solutions alone. Similar to the UV-Vis absorption results, conjugating CHP had no significant effect on the PA spectra of ICG@BSA NPs (Fig. 2F). The obtained spectra serve as reference signatures for identifying these nanoparticles in subsequent in vivo PA imaging studies.

### Biosafety evaluation of ICG@BSA-CHP NPs

To evaluate the biodistribution and potential toxicity of ICG@BSA-CHP NPs, we performed fluorescence (FL) images of ex vivo main organs from ApoE-/- mice at 1, 6, 12, and 24 h following intravenous injection of the NPs. The FL images revealed that after injection, the ICG@BSA-CHP NPs initially showed strong fluorescence signals, particularly in the liver and kidneys. Over time, these signals diminished due to excretion via hepatic metabolism, with fluorescence almost entirely cleared within 24 h (Figure S1A and B). These findings suggested that the ICG@BSA-CHP NPs were efficiently cleared from the body within a relatively short time, minimizing the risk of long-term toxicity.

Additionally, we conducted hematological analysis and H&E staining of the main organs (heart, liver, spleen, lung and kidney) from the ApoE-/- mice at 1 day, 7 days and 14 days after the intravenous injection of the ICG@BSA-CHP NPs. As listed in Figure S1C, no significant changes of the hemocyte test and functions of liver and kidneys were observed after injection. Moreover, the H&E staining results demonstrated that the tissues maintained normal structures and no obvious organ damage or inflammatory lesions were observed after injection (Figure S1D). Overall, these results collectively indicated that ICG@BSA-CHP NPs exhibited the good biosafety profiles in ApoE-/- mice, supporting their potential for further in vivo experiments.

### In vivo PA imaging and ex vivo FL imaging

**Fig. 3:**
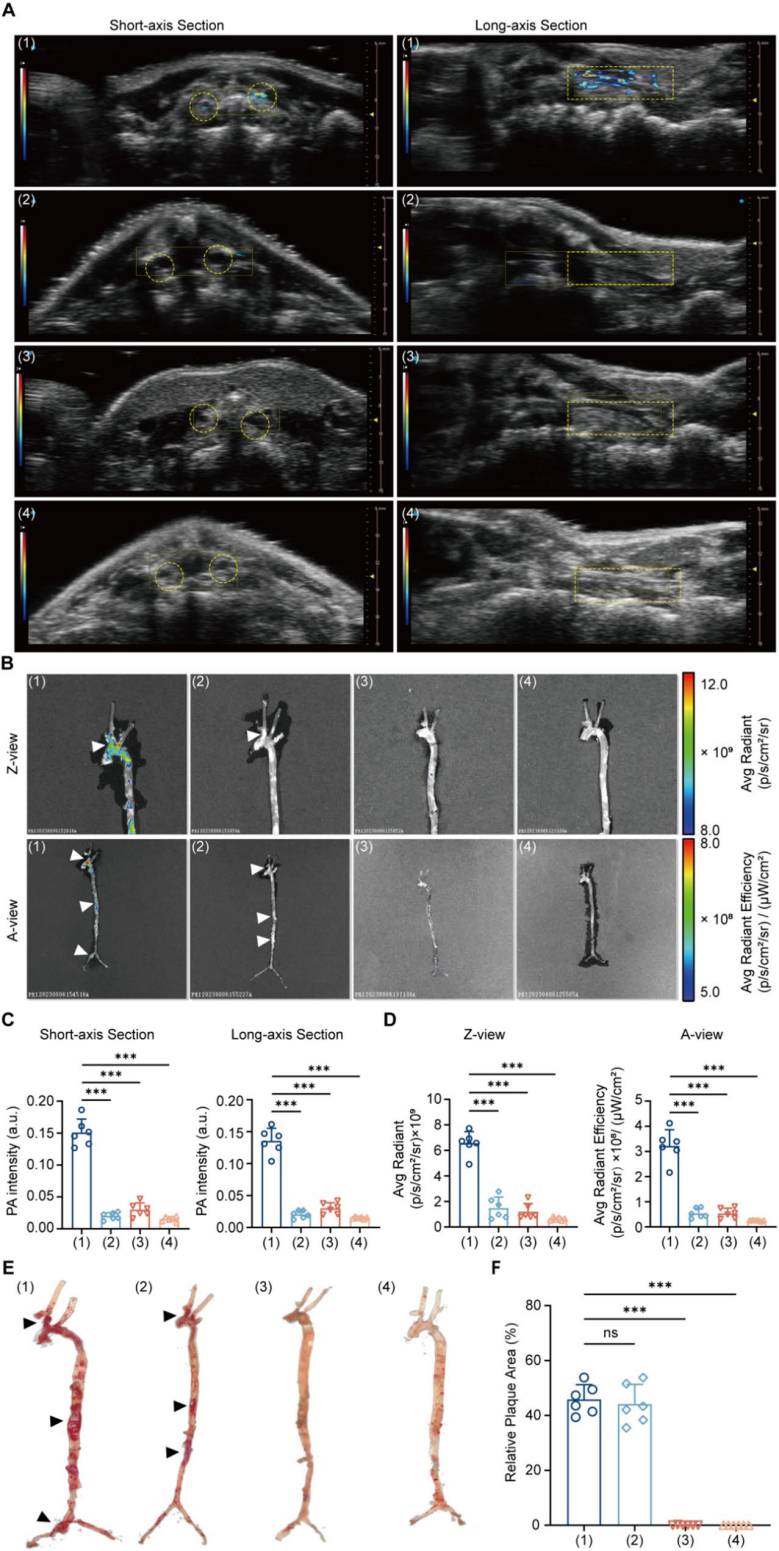
In vivo and Ex vivo Imaging. Experimental mice were divided into four groups according to different treatments: (1) AS mice model intravenously injected with ICG@BSA-CHP NPs; (2) AS mice model intravenously injected with ICG@BSA NPs; (3) Control mice model intravenously injected with ICG@BSA-CHP NPs; (4) Control mice model intravenously injected with ICG@BSA NPs. **A**) In vivo PA images of both short- and long-axis sections of the carotid artery for each group. Carotid arteries indicated by yellow circle (short-axis section) and rectangle (long-axis section). **B**) Ex vivo fluorescence imaging of the harvested aorta and carotid artery were captured in Z-view and A-view. Significant regions of atherosclerotic plaque indicated by white arrowheads. Quantitative statistics of in vivo PA intensity **C**) and ex vivo FL intensity **D**) of different groups. **E**) ORO staining photographs of the aorta and carotid arteries of the experimental mice, with significant atherosclerotic plaque regions showed by black arrowheads. **F**) Quantitative statistics of plaque areas in the different groups. Data in (**C**, **D**, **F**) were expressed as mean ± SD. *P < 0.05, **P < 0.01, ***P < 0.001; ns, no significance

After constructing the PA NPs, atherosclerotic mice were anesthetized and subjected to PA imaging for in vivo atherosclerotic plaque imaging. Considering occlusion avoiding and multidimensional evaluation, we collected ultrasonic (US) and PA images from both short-axis and long-axis perspectives (Fig. 3A).

Upon intravenous injection of ICG@BSA-CHP NPs, the carotid arteries of atherosclerotic (AS) mice model exhibited a significantly stronger PA signal compared to control mice, both in the short axis and long axis views (short axis: AS mice vs. Control mice, 0.15 ± 0.02 vs. 0.03 ± 0.01, *n* = 6, *P* < 0.001; long axis: AS mice vs. Control mice, 0.14 ± 0.02 vs. 0.03 ± 0.01, *n* = 6, *P* < 0.001). This enhanced PA signal indicated that ICG@BSA-CHP NPs effectively differentiate between atherosclerotic and normal arteries, likely due to their targeting ability for atherosclerotic plaques. Additionally, when comparing AS mice model injected with ICG@BSA-CHP NPs to those injected with ICG@BSA NPs, the former showed a markedly higher PA signal (short axis: ICG@BSA-CHP NPs vs. ICG@BSA NPs, 0.15 ± 0.02 vs. 0.02 ± 0.01, *n* = 6, *P* < 0.001; long axis: AS mice vs. Control mice, 0.14 ± 0.02 vs. 0.02 ± 0.01, *n* = 6, *P* < 0.001). This result indicated that the incorporation of CHP into the nanoparticles improved targeting and recognition of atherosclerotic plaques (Fig. 3C).

To further validate the specificity of PA signals for atherosclerotic plaques, ex vivo FL was performed on isolated aorta and carotid arteries, followed by ORO staining. Consistent with PA imaging results, AS mice model injected with ICG@BSA-CHP NPs showed significantly higher FL signals compared to other groups in carotid arteries (A view : AS mice + ICG@BSA-CHP NPs vs. AS mice + ICG@BSA NPs vs. Control mice + ICG@BSA-CHP NPs vs. Control mice + ICG@BSA NPs, 3.22 ± 0.64 vs. 0.54 ± 0.19 vs. 0.54 ± 0.20 vs. 0.26 ± 0.24, *n* = 6, *P* < 0.001; Z view : AS mice + ICG@BSA-CHP NPs vs. AS mice + ICG@BSA NPs vs. Control mice + ICG@BSA-CHP NPs vs. Control mice + ICG@BSA NPs, 6.50 ± 0.92 vs. 1.50 ± 0.85 vs. 1.22 ± 0.62 vs. 0.64 ± 0.14, *n* = 6, *P* < 0.001) (Fig. 3B and D). This enhanced fluorescence indicated that CHP enhances nanoparticle enrichment in atherosclerotic plaque regions due to its targeting capability.

Furthermore, the alignment between FL imaging and ORO staining revealed that the fluorescence accumulation regions (white arrowheads) closely matched the dye accumulation regions observed in the ORO staining images (black arrowheads) in the carotid artery, aortic arch, and thoraco-abdominal aortic junction. This correspondence further indicated that ICG@BSA-CHP NPs effectively realized atherosclerotic plaques in vivo and in vitro (Fig. 3B and F).

### Preparation of therapeutic NPs

In alignment with the preparation of PA NPs, we used PTX@HSA NPs as the primary therapeutic agent. CHP was then conjugated to the PTX@HSA NPs via a copper-free click chemical reaction as described previously.

**Fig. 4:**
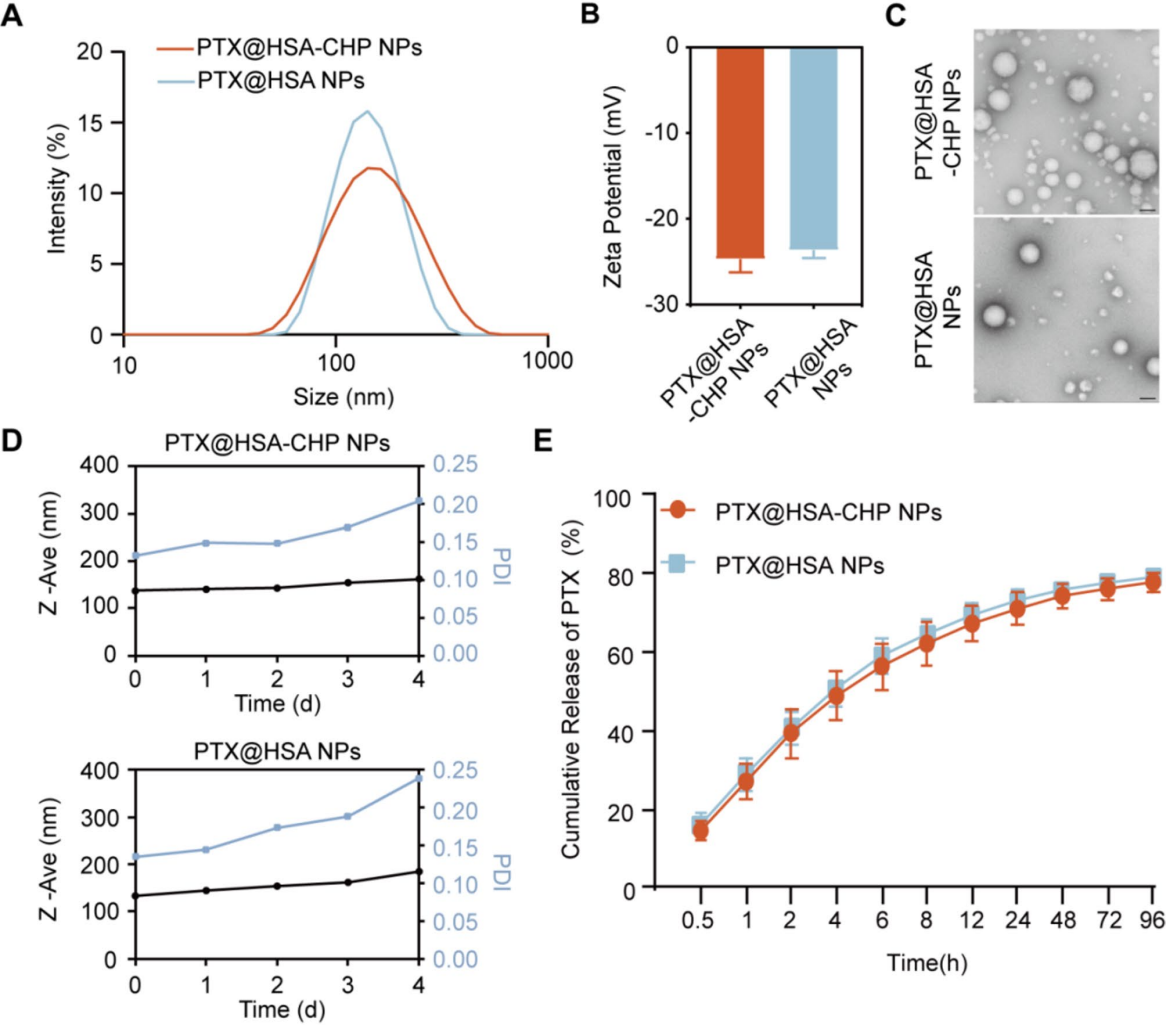
Characterizations of therapeutic albumin NPs. **A**) Particle Size distribution and **B**) Zeta potential of PTX@HSA-CHP NPs and PTX@HSA NPs. **C**) Morphology of NPs observed by TEM. The scale bars were 100 nm. **D**) Physical Stability of the NPs were measured by DLS. **E**) In vitro release of PTX from PTX@HSA-CHP NPs and PTX@HSA NPs

The particle sizes of PTX@HSA-CHP NPs and PTX@HSA NPs were measured using DLS, revealing sizes of 136.7 nm (PDI = 0.192) and 133.1 nm (PDI = 0.125), respectively (Fig. 4A). The zeta potentials of PTX@HSA-CHP NPs and PTX@HSA NPs were − 24.67 mV and − 23.57 mV, respectively (Fig. 4B). Transmission electron microscopy (TEM) images confirmed that both PTX@HSA-CHP NPs and PTX@HSA NPs maintained spherical shapes (Fig. 4C).

Long-term monitoring of the hydrodynamic diameter demonstrated that the NPs retained a consistent particle size over 4 days. However, beyond this period, the PDI increased and exceeded 0.2, potentially due to nanoparticle agglomeration, which is a common issue with albumin-based nanoparticles. Notably, the incorporation of CHP did not cause significant changes in DLS measurements compared to PTX@HSA NPs, indicating that CHP conjugation did not adversely affect the stability of the NPs (Fig. 4D). Additionally, in vitro drug release study showed similar cumulative release profiles for both PTX@HSA-CHP NPs and PTX@HSA NPs, suggesting that the addition of CHP did not significantly alter the drug release kinetics of the NPs (Fig. 4E).

### Biosafety evaluation of therapeutic NPs

To evaluate the biodistribution and potential toxicity of PTX@HSA-CHP NPs, we performed HPLC analysis to quantify PTX concentration in the major organs of ApoE-/- mice at 1, 6, 12, and 24 h following intravenous injection of the PTX@HSA-CHP NPs. The results showed that PTX initially accumulated predominantly in the liver and kidney, reaching peak levels at 6 h post-injection. Over time, the PTX concentration levels decreased due to hepatic metabolism and subsequent excretion, with nearly clearance observed within 24 h (Figure S2A). These findings suggested that PTX was mainly cleared via hepatic metabolism after release from NPs.

In addition, we conducted hematological analysis and H&E staining of the main organs (heart, liver, spleen, lung and kidney) from the healthy ApoE-/- mice at 1 day, 7 days and 14 days after the intravenous injection of the PTX@HSA-CHP NPs. As listed in Figure S2B, no significant changes of the hemocyte test and functions of liver and kidneys were observed after injection. Furthermore, H&E staining demonstrated that the tissues retained normal structures, with no observable organ damage or inflammatory lesions after treatment (Figure S2C). Collectively, these results indicated that PTX@HSA-CHP NPs exhibited a favorable biosafety profile in ApoE-/- mice, supporting their potential for further in vivo experiments.

### Evaluation of targeted delivery capability and therapeutic effect in vivo

**Fig. 5:**
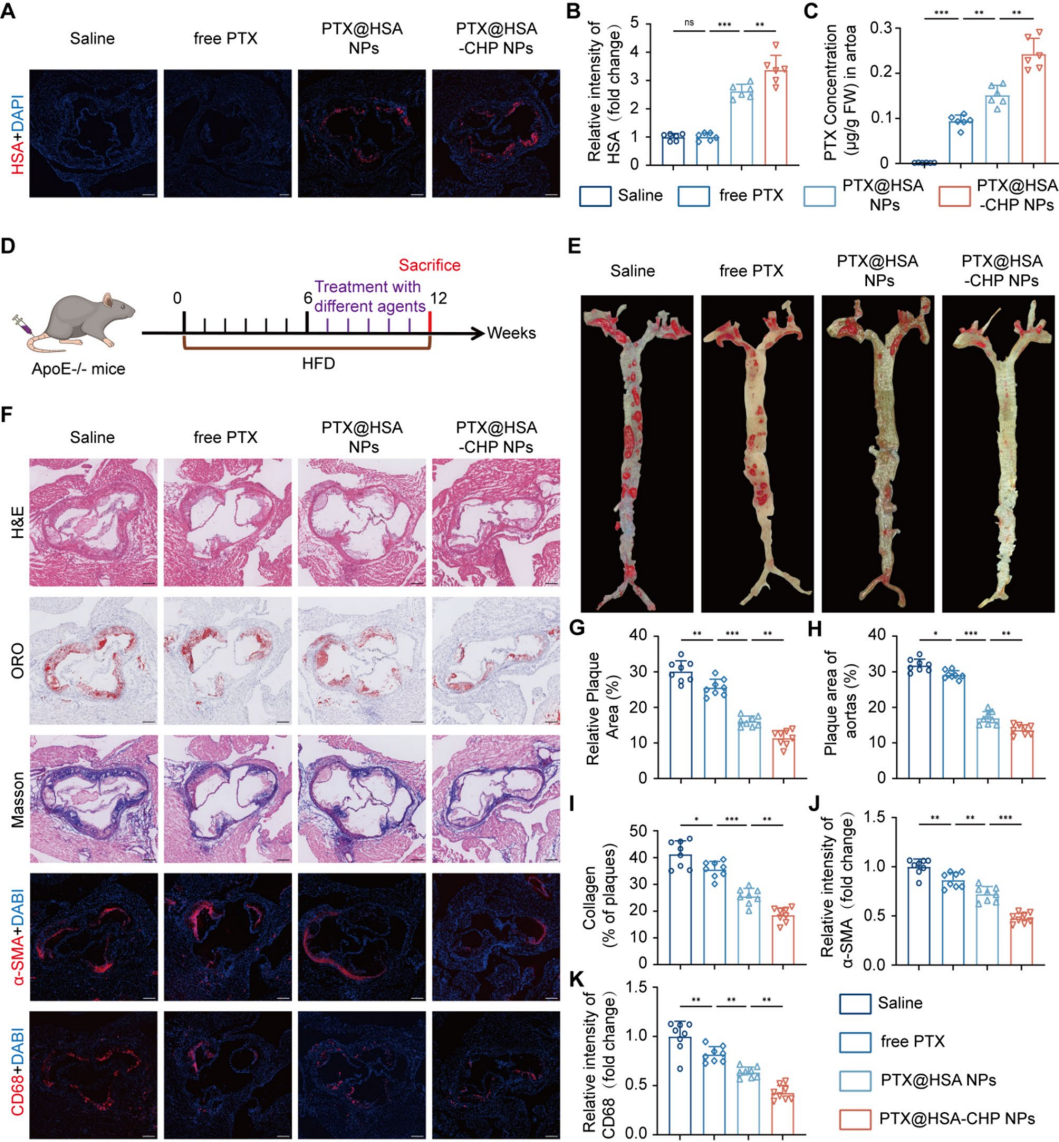
Evaluation of targeted drug delivery and therapeutic efficacy in vivo. **A**) Representative immunofluorescence images of atherosclerotic plaques, assessing albumin accumulation in plaques. **B**) Quantitative analysis of relative intensity of HSA in atherosclerotic plaques. **C**) Measurement of paclitaxel content within plaques. **D**) Schematic diagram of the therapeutic schedule. **E**) ORO staining of isolated aorta. **F**) Histological staining including H&E, ORO and Masson staining, and immunofluorescence staining including α-SMA and CD68 of aortic root. Quantitative analysis of relative plaque area of the **G**) aorta and **H**) aortic root, and **I**) collagen of plaques. Quantitative analysis of the fluorescence intensity of **J**) α-SMA and **K**) CD68 in aortic roots. Experimental data were expressed as mean ± SD. *P < 0.05, **P < 0.01, ***P < 0.001; ns, no significance. The scale bars were 200 μm

To evaluate the targeted delivery capability of the NPs, atherosclerotic mice, which had been fed with a high-fat diet (HFD) for 6 weeks, were treated with saline, free PTX, PTX@HSA NPs, and PTX@HSA-CHP NPs, respectively. Immunofluorescence staining results revealed that the PTX@HSA-CHP NPs group exhibited the strongest fluorescence signals within atherosclerotic plaques in the murine aorta (PTX@HSA-CHP NPs vs. PTX@HSA NPs, 3.38 ± 0.52 vs. 2.62 ± 0.24, *n* = 6, *P* < 0.01; PTX@HSA NPs vs. free PTX, 2.62 ± 0.24 vs. 1.00 ± 0.13, *n* = 6, *P* < 0.001; free PTX vs. Saline, 1.00 ± 0.13 vs. 1.00 ± 0.12, *n* = 6, *P* > 0.05) (Fig. 5A and B). Consistently, HPLC analysis showed that the concentration of paclitaxel in the aortas of the PTX@HSA-CHP NPs group was significantly higher than other groups, despite administering the same dose of paclitaxel (PTX@HSA-CHP NPs vs. PTX@HSA NPs, 0.2425 ± 0.0351 vs. 0.1512 ± 0.0219, *n* = 6, *P* < 0.01; PTX@HSA NPs vs. free PTX, 0.1512 ± 0.2189 vs. 0.0937 ± 0.0138, *n* = 6, *P* < 0.01; free PTX vs. Saline, 0.0937 ± 0.0138 vs. 0.0015 ± 0.0003, *n* = 6, *P* < 0.001) (Fig. 5C). These findings demonstrated that the conjugation with CHP markedly enhanced the plaque targeting drug delivery capability of albumin NPs, making them a promising strategy for targeted treatment of atherosclerosis.

To further evaluate the therapeutic efficacy of the NPs, AS mice fed with a HFD for 6 weeks were randomly divided into four groups: saline, free PTX, PTX@HSA NPs, and PTX@HSA-CHP NPs. Each group received weekly intravenous injections of the respective treatments for 6 weeks (Fig. 5D). Compared to the other group, AS mice treated with PTX@HSA-CHP NPs exhibited significantly reduced aortic plaque areas (PTX@HSA-CHP NPs vs. PTX@HSA NPs, 11.36 ± 2.15 vs. 16.09 ± 1.44, *n* = 8, *P* < 0.01; PTX@HSA NPs vs. free PTX, 16.09 ± 1.44 vs. 25.58 ± 2.35, *n* = 8, *P* < 0.001; free PTX vs. Saline, 25.58 ± 2.35 vs. 30.13 ± 2.98, *n* = 8, *P* < 0.01), indicating the notably therapeutic effect (Fig. 5E and G).

At the same time, comparison of ORO-stained frozen sections of the aortic root supported these findings with a similar trend (Fig. 5F). It could be seen that the PTX@HSA-CHP NPs treatment group exhibited the lowest percentage of plaque area (PTX@HSA-CHP NPs vs. PTX@HSA NPs, 13.65 ± 1.38 vs. 16.96 ± 1.87, *n* = 8, *P* < 0.01; PTX@HSA NPs vs. free PTX, 16.96 ± 1.87 vs. 29.13 ± 1.17, *n* = 8, *P* < 0.001; free PTX vs. Saline, 29.13 ± 1.17 vs. 31.80 ± 1.75, *n* = 8, *P* < 0.05) (Fig. 5H). Furthermore, collagen content within the plaques, identified by Masson staining, showed that mice injected with PTX@HSA-CHP NPs had the least collagen deposition compared to the other groups (PTX@HSA-CHP NPs vs. PTX@HSA NPs, 18.52 ± 2.71 vs. 25.53 ± 3.00, *n* = 8, *P* < 0.01; PTX@HSA NPs vs. free PTX, 25.53 ± 3.00 vs. 35.56 ± 3.01, *n* = 8, *P* < 0.001; free PTX vs. Saline, 35.56 ± 3.01 vs. 41.28 ± 4.98, *n* = 8, *P* < 0.05) (Fig. 5F and I). Meanwhile, owing to the antiproliferative and anti-inflammatory function of paclitaxel, the relative fluorescence intensity of α-SMA and CD68 were significantly decreased in both the PTX@HSA NPs and PTX@HSA-CHP NPs groups compared to the saline and free PTX group (α-SMA : PTX@HSA-CHP NPs vs. PTX@HSA NPs, 0.48 ± 0.05 vs. 0.72 ± 0.08, *n* = 8, *P* < 0.001; PTX@HSA NPs vs. free PTX, 0.72 ± 0.08 vs. 0.86 ± 0.08, *n* = 8, *P* < 0.01; free PTX vs. Saline, 0.86 ± 0.08 vs. 1.00 ± 0.08, *n* = 8, *P* < 0.01; CD68: PTX@HSA-CHP NPs vs. PTX@HSA NPs, 0.43 ± 0.08 vs. 0.64 ± 0.05, *n* = 8, *P* < 0.01; PTX@HSA NPs vs. free PTX, 0.64 ± 0.05 vs. 0.82 ± 0.08, *n* = 8, *P* < 0.01; free PTX vs. Saline, 0.82 ± 0.08 vs. 1.00 ± 0.15, *n* = 8, *P* < 0.01) (Fig. 5F). Notably, PTX@HSA-CHP NPs exhibited superior anti-smooth muscle cell proliferation ability and the lowest degree of macrophage infiltration, consistent with the treatment effects observed in the ORO and H&E staining (Fig. 5J and K). Taken together, these results highlighted that the conjugation of CHP significantly enhanced the therapeutic effect of the PTX@HSA NPs by improving the targeted drug delivery ability, underscoring its potential for improved treatment of atherosclerosis.

## Discussion

In this study, we demonstrated that CHP binding is highly specific against atherosclerotic plaques in both human and mice artery. Additionally, we successfully developed CHP-functionalized photoacoustic nanoparticles capable of targeting degraded collagen in atherosclerosis mouse model, enabling precise imaging of plaque regions. Furthermore, we modified the clinically applied PTX@HSA NPs by conjugating CHP, which has been shown to significantly improve the delivery of paclitaxel to atherosclerotic plaques and effectively inhibited atherosclerotic plaque progression without causing noticeable toxicity.

Collagen synthesis and degradation are essential for the development, progression, and clinical manifestations of atherosclerosis [20]. A recent study revealed that in ApoE-/- mice fed with a high-fat diet, collagen degradation detected by CHP primarily occurs within atherosclerotic plaques, and increases in parallel with the progression of atherosclerosis [21]. Similarly, we validated the detection capability of CHP in mice arterial tissues and found that collagen degradation, as identified by CHP, was also pronounced in human atherosclerotic arterial tissues, with a particular concentration in plaque regions.

CHP, as a synthetic collagen mimetic peptide, can specifically binds to degraded collagen strands by reforming a new hybrid triple-helix structure [17, 22]. Previous studies have shown that CHP can be utilized to detect collagen degradation in various diseases including intervertebral disc degeneration, subretinal fibrosis and achilles tendinopathy [23–27]. Additionally, Chen et al. enhanced antimicrobial efficacy and facilitated tissue repair in a rat wound healing model by conjugating CHP [19]. In recent years, copper-free click chemistry strategies has been widely utilized in nanoparticle drug construction and delivery systems due to its high specificity and efficiency, mild reaction conditions and minimal cytotoxicity [28–30]. In the present study, we synthesized a CHP sequence with an azide group and modified albumin nanoparticles with DBCO-NHS. The copper-free click chemistry between DBCO and the azide group enabled the assembly of the CHP peptide into the albumin NPs.

ICG, approved by the United States Food and Drug Administration (FDA), has been widely used as a photoacoustic (PA) imaging dye in various studies due to its low toxicity, good light stability and excellent biocompatibility [31–33]. Recent studies have demonstrated that self-assembling ICG with BSA into nanoparticles can enhance its near-infrared fluorescence and improve the guidance of in vivo cancer photodynamic therapy [34–36]. In this study, we utilized BSA NPs encapsulating ICG as a contrast agent for PA imaging. Our results demonstrated that only imaging probes containing CHP could effectively identify atherosclerotic plaques in PA imaging. In contrast, probes without CHP failed to highlight the plaques due to their lack of targeting ability. ICG@BSA-CHP NPs presented a novel approach for the molecular imaging of atherosclerotic plaques and showed great potential for clinical applications, such as intravascular photoacoustic imaging.

Currently, antiproliferative drugs like paclitaxel are widely used in intraluminal drug coated devices for atherosclerosis [9, 37]. However, the lack of targeting delivery, drug loss and rapid metabolism contribute to suboptimal therapeutic outcomes [38]. PTX@HSA NPs, as an FDA approved commercial nanomedicine, has been successfully implemented in clinical practice for the treatment of various proliferative diseases [39, 40]. Compared to conventional paclitaxel, PTX@HSA NPs offer a greater distribution volume, higher drug concentration and prolonged release [41]. Additionally, PTX@HSA NPs enhance transendothelial transport by increasing endothelial transcytosis through the gp60 albumin receptor/caveolin-1 pathway, thereby improving drug delivery efficiency [42, 43]. In this study, we employed a copper-free click chemistry reaction to functionalize PTX@HSA NPs with CHP, creating a nanodrug specifically targeting atherosclerotic plaques. Our results demonstrated that PTX@HSA-CHP NPs significantly enhanced plaque targeting ability and effectively inhibited the progression of atherosclerotic plaques without obvious toxicity, compared to the PTX@HSA NPs. Therefore, we anticipated that the strategy of conjugating CHP to PTX@HSA NPs could open new avenues for current endovascular treatment of atherosclerosis and provide a new solution for molecular therapy of atherosclerotic plaques.

## Conclusion

In summary, we demonstrated that collagen hybrid peptide (CHP) effectively targeted atherosclerotic plaque by specifically binding to the degraded collagen during atherosclerosis progression. Leveraging copper-free click chemistry, we developed CHP-functionalized albumin nanoparticles by integrating CHP into ICG@BSA imaging NPs and PTX@HSA therapeutic NPs. Comprehensive characterization confirmed that CHP conjugation minimally impacted the physicochemical properties of these nanoparticles. In vivo experiments using a mice model revealed that CHP functionalized nanoparticles significantly enhanced both imaging ability and therapeutic efficacy without eliciting notable toxicity. These findings indicated that CHP, as a synthetic peptide, markedly enhanced the targeting capability and effectiveness of the mature imaging and therapeutic nanoparticles. Consequently, CHP functionalized albumin nanoparticles could realize the targeted identification and treatment of atherosclerotic plaques, which was expected to provide a promising solution for the future atherosclerosis molecular imaging and treatment.

## Supplementary Information

Supplementary Material 1.

## Data Availability

No datasets were generated or analysed during the current study.
