# Supplementary material for: Enhanced atherosclerosis molecular imaging and therapy with collagen hybridizing peptide functionalized albumin nanoparticles
Source: J Nanobiotechnology. 2025 Oct 28;23:691. doi: 10.1186/s12951-025-03721-3 (PMC12560523; doi:10.1186/s12951-025-03721-3)
Supplement: Supplementary file 1 — Supplementary Material 1. [file 12951_2025_3721_MOESM1_ESM.docx]

**Appendix A. Supplementary data**

**Enhanced atherosclerosis molecular imaging and therapy with collagen hybridizing peptide functionalized albumin nanoparticles**

Table S1. The groups imformation for in vivo PA imaging and ex vivo FL imaging

| Groups | Numbers | Experiment timelines | Treatment |
| --- | --- | --- | --- |
|  | 6 | 6 - 8 weeks age ApoE-/- mice fed with HFD for 12 weeks, and then sacrifice for experiments | AS mice model intravenously injected with ICG@BSA-CHP NPs (800 μM) |
|  | 6 |  | AS mice model intravenously injected with ICG@BSA NPs (800 μM) |
|  | 6 | 6 - 8 weeks age ApoE-/- mice fed with normal diet for 12 weeks, and then sacrifice for experiments | Control mice model intravenously injected with ICG@BSA-CHP NPs (800 μM) |
|  | 6 |  | Control mice model intravenously injected with ICG@BSA NPs (800 μM) |

Table S2. The groups imformation for targeted delivery capability of NPs

| Groups | Numbers | Experiment timelines | Treatment |
| --- | --- | --- | --- |
| Saline | 6 | 6 - 8 weeks age ApoE-/- mice fed with HFD for 6 weeks, and then sacrifice for experiments | treated with saline |
| free PTX | 6 |  | treated with free PTX (equivalent to PTX 0.25 mg/kg) |
| PTX@HSA NPs | 6 |  | treated with PTX@HSA NPs (equivalent to PTX 0.25 mg/kg) |
| PTX@HSA-CHP NPs | 6 |  | treated with PTX@HSA-CHP NPs (equivalent to PTX 0.25 mg/kg) |

Table S3. The groups imformation for therapeutic effect of NPs

| Groups | Number of samples | Experiment timelines | Treatment |
| --- | --- | --- | --- |
| Saline | 8 | 6 - 8 weeks age ApoE-/- mice fed with HFD for 12 weeks,during this period the mice were adminstrated with different threatment in the last 6 weeks | treated with saline once per week |
| free PTX | 8 |  | treated with free PTX (equivalent to PTX 0.25 mg/kg) once per week |
| PTX@HSA NPs | 8 |  | treated with PTX@HSA NPs (equivalent to PTX 0.25 mg/kg) once per week |
| PTX@HSA-CHP NPs | 8 |  | treated with PTX@HSA-CHP NPs (equivalent to PTX 0.25 mg/kg) once per week |


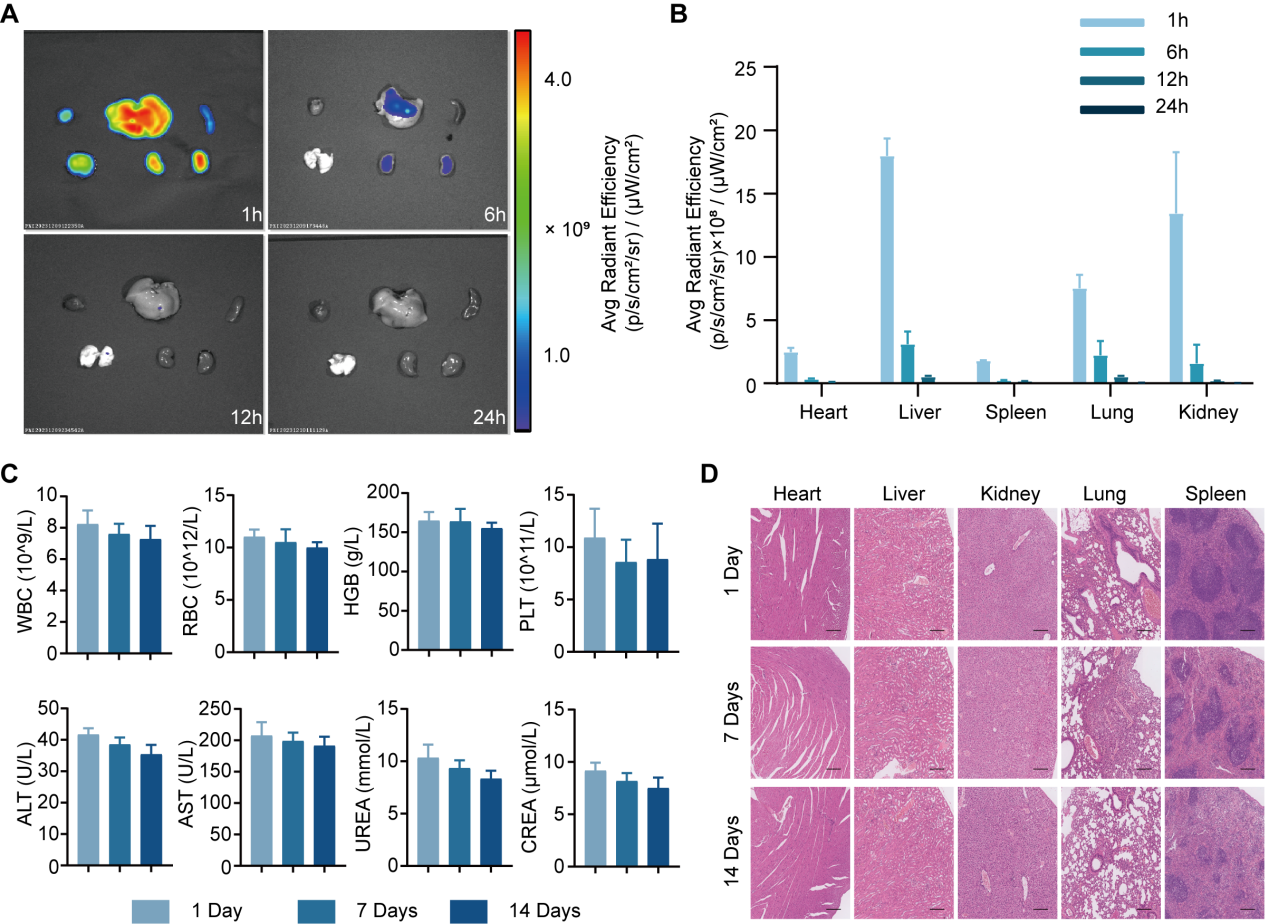


**Figure S1.** Biosafety evaluation of ICG@BSA-CHP NPs. A) FL biodistribution after the intravenous injection of ICG@BSA-CHP NPs. B) Quantitative analysis of FL intensity in different time points. C) The blood routine examination and blood biochemistry test of healthy ApoE-/- mice after intravenous injection of ICG@BSA-CHP NPs for different days. WBC: white blood cells; RBC: red blood cells; HGB: hemoglobin; PLT: platelets; ALT: aminotransferase; AST: aspertate aminotransferase; UREA: urea; CREA: creatinine. D) Histological analysis of the major organs of mice after injection of ICG@BSA-CHP NPs for 1 day, 7 days and 14 days, respectively. All the scale bars are 200 μm.


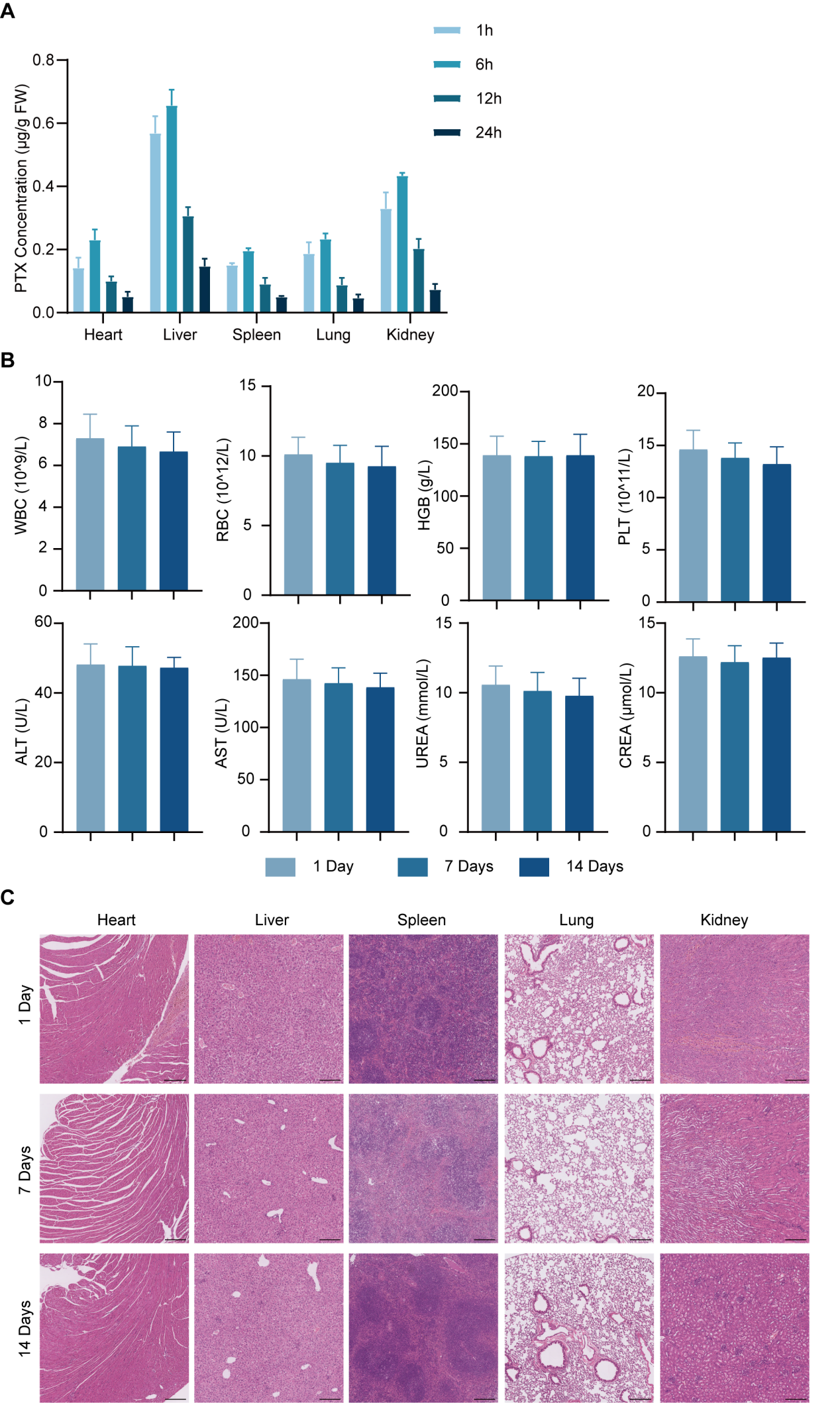


**Figure S2.** Biosafety evaluation of ICG@BSA-CHP NPs. A) HPLC analysis of PTX biodistribution in main organs after the intravenous injection of PTX@HSA-CHP NPs at 1h, 6h, 12h, and 24h. B) The blood routine examination and blood biochemistry test of healthy ApoE-/- mice after intravenous injection of PTX@HSA-CHP NPs for 1 day, 7 days and 14 days, respectively. WBC: white blood cells; RBC: red blood cells; HGB: hemoglobin; PLT: platelets; ALT: aminotransferase; AST: aspertate aminotransferase; UREA: urea; CREA: creatinine. C) Histological analysis of the major organs of mice after injection of PTX@HSA-CHP NPs for different days. All the scale bars are 200 μm.
